# Thermodynamic Properties of Thorium Dioxide From 298 to 1,200 °K

**DOI:** 10.6028/jres.065A.013

**Published:** 1961-04-01

**Authors:** Andrew C. Victor, Thomas B. Douglas

## Abstract

As a step in developing new standards of heat capacity applicable up to very high temperatures, the heat content (enthalpy) of thorium dioxide, ThO_2_, relative to 273 °K, was accurately measured at ten temperatures from 323 to 1,173 °K. A Bunsen ice calorimeter and a drop method were used to make the measurements on two samples of widely different bulk densities. The corresponding heat-capacity values for the higher density sample are represented within their uncertainty (estimated to be ±0.3 to 0.5%) by the following empirical equation 1 (cal mole^−1^ deg^−1^ at T °K):

$$C_{p}^{o}=17.057+18.06\left(10^{-4}\right)\;T-2.5166\;\left(10^{5}\right)/T^{2}$$

At 298 °K this equation agrees with previously reported low-temperature measurements made with an adiabatic calorimeter. Values of heat content, heat capacity, entropy, and Gibb’s free energy function are tabulated from 298.15 to 1,200 °K.

## 1. Introduction

Current practical and theoretical developments have increased the need for accurate heat capacities and related thermal properties at high temperatures, yet the values reported for the same material from different laboratories often show large differences. In some cases the precision is correspondingly poor, but in other cases the disagreement is so largely attributable to systematic errors that the availability of suitable heat standards to monitor the accuracy is almost imperative. It is one of the fundamental functions of NBS to develop such standards as the need arises.

A material chosen for such a standard must meet certain requirements. In the temperature range of its use it must exist in a definite reproducible physical state, have a fixed chemical composition, and not be subject to appreciable spontaneous decomposition. In addition it is highly desirable that it not react chemically with the gases of the atmosphere (oxygen, nitrogen, carbon dioxide, and water vapor) nor undergo appreciable fusion, volatilization, or transition. Its cost should not be prohibitive, and its heat capacity should not be too small.

*α*-Aluminum oxide (corundum) meets these specifications excellently up to approximately 1,800 °K, and was therefore recommended by the Third Calorimetry Conference, which met in 1948, as a high-temperature heat-capacity standard. The Bureau obtained and conditioned a highly pure sample of a synthetic grade of this substance, and established its heat capacity accurately from 10 to 1,200 °K [1];^2^ using new apparatus recently constructed, these measurements are soon to be extended up to approximately 1,800 °K.

However, at higher temperatures aluminum oxide is impractical as a heat standard, for it becomes increasingly volatile, and melts at approximately 2,300 °K. A more refractory solid is needed at these temperatures, and preferably a substance which could serve as a standard at the lower temperatures also. There are approximately a dozen well-known substances of definite chemical composition whose melting points lie above 2,800 °K. Of these, almost the only ones which appear to have all the above desirable properties are thorium dioxide, beryllium oxide, and magnesium oxide.

As a standard, thorium dioxide would have the minor practical disadvantage that many impurities commonly present in it have specific heats which are considerably higher than its own. The latter fact causes the heat capacity per unit mass to be correspondingly sensitive to the exact chemical composition of the sample used. Advantages outweighing this fact, however, are the unusually high melting point of thorium dioxide (reported to be 3,300 °K [2]), its monomorphism, and its high chemical stability and inertness.

The temperatures to which beryllium oxide and magnesium oxide could be used as a standard are somewhat lower because their melting points (reported to be about 2,800 °K and 3,000 °K respectively [2]) are not as high. However, compared with thorium dioxide, their heat capacities per unit mass are much less sensitive to the influence of common impurities, and also, they are less expensive. The accurate knowledge of the heat capacities of all three substances over long temperature ranges would have added value because their favorable high-temperature properties lead to their very frequent use as structural materials in many installations operated at elevated temperatures.

Recent accurate measurements at the Bureau of the heat capacity of ThO_2_ from 298.15 to 1,200 °K are reported in this paper. The Bureau plans to extend these measurements up to 1,800 °K in the near future, and probably up to 2,800 °K within the next few years. Since it is difficult to melt such a refractory substance without incurring undesirable contamination, the present investigation was carried out on two pressed and sintered samples of widely different bulk densities, in order to test the sensitivity of the thermal values to variations in this property.

A paper describing similar measurements on beryllium oxide and magnesium oxide will be published shortly. The authors believe that these papers will present evidence supporting the advisability of higher temperature heat-content measurements on ThO_2_, BeO, and MgO, which may eventually lead to the adoption of one of them as a calorimetric standard at temperatures above 1,800 °K.

## 2. Samples and Containers

Each sample consisted of two cylinders, each 2 cm long and 1 cm in diameter, which were prepared in the Engineering Ceramics Section of the Bureau by pressing, firing and sintering fine powder of high purity. Variations of firing temperatures made it possible to obtain samples of different bulk densities so as to be able to investigate any existing dependence of heat capacity on density.

The thorium dioxide in the form of powder was supplied by Lindsay Chemical Company, of Chicago, Illinois. By firing to different high temperatures, samples of 9.7 g cm^−3^ and 7.2 g cm^−3^ bulk density were obtained. The density calculated from X-ray diffraction data is 10.04 g cm^−3^. These two samples have densities which are 97 and 72 percent of this density, and will hereafter be called thorium dioxide samples 1 and 2 respectively.

Specimens of each of the two densities of thorium dioxide were analyzed spectrochemically at the Bureau. The results of these analyses are given in table 1.^3^ The samples were analyzed for the 44 chemical elements listed in table 1, 30 of which were determined by the use of synthetic standards made from ThO_2_. Although the alkali metals, U, La, Ce, and Y were not detected, neither analytical standards for these elements nor the techniques of using them were available.

Assuming each element detected to be present in the form of its highest stable oxide, the thorium dioxide samples were calculated to be 99.95 percent ThO_2_ by weight.

Published values of the heat capacities of the contaminating oxides were used to correct, on an additive basis, all the heat measurements to the basis of pure ThO_2_. These heat measurements were sufficiently precise that application of the corrections for the impurities, which never exceeded 0.1 percent, should reduce the systematic error significantly.

The samples were enclosed in sealed containers of annealed pure silver (999.5 fine, cylinders 1.8 in. long and of ⅝ in. diam). After introducing the sample and enough extra silver to adjust the total final mass of this metal to a standard value (exactly 12 g), the protruding edges of the two end caps (each 0.02 in. thick), which were shaped to fit the cylinder, were flame-welded to the ends of the cylinder (wall thickness, 0.015 in.). Since the sample and container had an average temperature of approximately 700 °C when the final sealing was accomplished, the amount of air sealed in was insufficient to distort the container by internal pressure at the highest temperatures subsequently used. A Pt—10 percent Rh wire of known mass encircled a groove around the silver cylinder and served to suspend the latter in the furnace and calorimeter.

In accurate work, there are certain advantages in using containers composed of silver instead of a hard base metal such as 80 Ni–20 Cr, which the authors have often used. Unlike silver, the alloy mentioned undergoes a transition near 550 °C, shows small but detectable changes in heat capacity attributed to minor annealing effects, and through traces of oxidizing gases accumulates surface coatings of oxides that must be measured by frequent reweighing and be corrected for. The mechanically sealed base-metal containers frequently develop leaks. It appears that during the short time such a silver container is falling into the calorimeter the radiation at the highest temperatures, which must be constant to avoid error in the heat measurements, decreases slowly but measurably from run to run. This effect presumably occurs through grain growth and consequent lowering of the emissivity of the silver surfaces, but the error appears to be negligible if the container is conditioned by being held for several hours in the furnace at 900 °C before the heat measurements are begun. Silver containers sealed as described above would be inapplicable to samples which, unlike thorium dioxide, must be kept cold during the sealing to prevent volatilization or must be kept in an inert atmosphere to prevent reaction with air. This disadvantage could be avoided by substituting the superior sealing technique of induction welding.

## 3. Heat Content Measurements

The “drop” method employed in the heat-content measurements has been described in detail in a previous publication [1]. In brief, the method was as follows. The sample, sealed in a container, was suspended in a silver-core furnace until it had time to come to a constant known temperature. It was then dropped (with almost free fall) into the Bunsen ice calorimeter, which measured the heat evolved by the sample plus container in cooling to 0 °C. In order to account for the heat content of the container and the heat lost during the drop, a similar experiment was made with the empty container (or an identical one) at the same furnace temperature. The difference between the two values of heat is a measure of the heat-content change of the sample between 0 °C and the temperature in the furnace.

Up to and including 600 °C^4^ a strain-free platinum resistance thermometer was used to measure the temperature of the central portion of the furnace. A platinum—platinum-10 percent rhodium thermocouple was used over the whole temperature range of the furnace: below 600 °C it did not compete with the more precise resistance thermometer, but only served to detect any otherwise unsuspected large change in either measuring instrument. Above 600 °C the thermocouple alone was used.

Table 2 gives the measured heat values obtained in individual runs for the empty container in calories (one defined calorie being equivalent to 4.1840 joules). The chronological order of the furnace temperatures for the experiments was as follows: 900°, 700°, 100°, 200°, 300°, 500°, 600°, 800°, 400°, 50°. The 900 °C experiments were made first in order to condition the surface of the capsule so that its emissivity, and hence its heat loss during a drop into the calorimeter, would be little affected by subsequent heat treatment. Results on another container (having the same masses of parts), which were obtained three years earlier, were higher than those of table 2 by 0.6 cal at 100 and 200 °C. However, at and above 300 °C there was no systematic difference between the two sets, the average discrepancy in this temperature range being only ±0.12 cal. The earlier container was run at all temperatures without preliminary heat treatment at 900 °C. This agreement, as well as the conditioning of the present empty container before its use, increased the authors’ confidence in the reproducibility of the empty-container heat values. Since in determining the heat content of the samples it was not convenient to use the same containers as those in which the samples were measured, it was important to verify the assumption that this substitution of containers caused inappreciable error.

In table 3, the second and third columns contain fully corrected heat-content values, in defined calories per gram, for thorium dioxide samples 1 and 2 (masses 37.1217 g and 22.8781 g respectively). These values were obtained with the furnace temperatures in random order. The listed individual heat contents were obtained by subtracting the empty container values, using the mean at each temperature, from the observed heat contents for sample plus container and dividing the resulting heat contents by the sample mass. Corrections had been applied for the calorimeter heat leak, the deviations of the sample container from standard conditions, weighing in air instead of vacuum, the extra air in the empty container because no sample was present, and for the heat contributions of analyzed impurities in the sample as described in section 2. The largest correction, that for impurities, was −0.1 ±0.005 percent of the net heat content of the sample at all temperatures. The sum of all other corrections amounts to approximately 0.01 percent of the net heat content. The calculated values of heat content in table 3 are smoothed values arrived at as described in section 4.

## 4. Smoothed Thermodynamic Functions

The mean heat content values in table 3, column 4, are for ThO_2_ sample 1 alone. Because this sample is closer in density to the X-ray value, and hence presumably a more reproducible state, than sample 2, the authors felt that the values from this sample alone would be more meaningful. Using the method of least squares, the values in column 4 were used to determine the constants in eq (1) for thorium dioxide (in cal g^−1^ at *t* °C):

$$\begin{array}{c} H_{t}^{0}-H_{0^{{}^{\circ}}C}^{0}=0.0664596\\ +3.418\left(10^{-6}\right)t^{2}-3.488t/\left(t+273.15\right) \end{array}$$ (1)

This form of equation was found to fit the low-temperature measurements (50 and 100 °C) better than an equation with the third term proportional to log_10_ [(*t*+273.15)/273.15], which has commonly been used in representing high-temperature metal-oxide heat contents obtained in this laboratory.

Using the following thermodynamic relations

$$C_{p}^{o}={\left(\partial H/\partial T\right)}_{p}$$ (2)

$$S_{T}^{o}-S_{0^{{}^{\circ}}K}^{o}=\int_{0}^{T}C_{p}^{o}\;dT/T$$ (3)

$$-\left(F_{T}^{o}-H_{0^{{}^{\circ}}K}^{o}\right)/T=S_{T}^{o}-S_{0^{{}^{\circ}}K}^{o}-\left(H_{T}^{o}-H_{0^{{}^{\circ}}K}^{o}\right)/T$$ (4)

equations were derived from eq (1) for the molar heat content, heat capacity, entropy, and Gibbs-free-energy function for ThO_2_ (molecular weight, 264.12). The values of thermodynamic functions at 298.15 °K were evaluated from Osborne and Westrum’s values for $\left(H_{298.15}^{o}-H_{0^{{}^{\circ}}K}\right)$ and $\left(S_{298.15}^{o}-S_{0^{{}^{\circ}}K}\right)$ [4]. Osborne and Westrum’s measurements on ThO_2_ (10 to 305 °K) gave heat-capacity values identical at 298.15° and different (higher) by only 0.3 percent at 273.15 °K than those given by eq (1). Furthermore, their adiabatic calorimeter has shown excellent agreement (in general, within ±0.1% in heat capacity) with Bureau adiabatic calorimeters, as demonstrated by work in both laboratories on standard-sample benzoic acid [5, 6]. Their heat capacities of ThO_2_ are believed to be more reliable between 273° and 298 °K than those given by eq (1). The following final equations for ThO_2_ (in terms of cal mole^−1^ at *T* °K and one atm pressure) may therefore be considered strictly applicable only over the temperature range 298°–1,200 °K:

$$H_{T}^{o}-H_{0^{{}^{\circ}}K}^{o}=17.0601\;T+9.028\left(10^{-4}\right)\;T^{2}+2.5166\left(10^{5}\right)/T-3486.55$$ (5)

$$C_{p}^{o}=17.060+18.06\left(10^{-4}\right)T-2.5166\left(10^{5}\right)/T^{2}$$ (6)

$$S_{T}^{o}-S_{0^{{}^{\circ}}K}^{o}=39.2826\;\log_{10}\;T+18.056\left(10^{-4}\right)T+1.2583\left(10^{5}\right)/T^{2}-83.5645$$ (7)

$$-\left(F_{T}^{o}-H_{0^{{}^{\circ}}K}^{o}\right)/T=39.2826\;\log_{10}\;T+9.028\left(10^{-4}\right)T-1.2583\left(10^{5}\right)/T^{2}+3486.55/T-100.6246$$ (8)

Values for ThO_2_ calculated from eqs (5) to (8) at selected temperatures are given in table 4.

**Figure 1:**
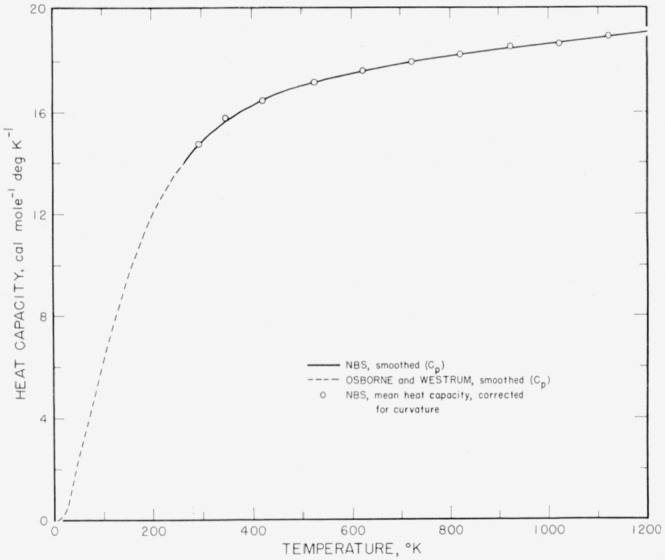
Heat capacity of thorium dioxide.

Figure 1 represents the heat capacity from 0 to 1,200 °K. The two independent curves for *C_p_* represent the smooth values of Osborne and Westrum and of the present work respectively, and join very smoothly, as noted above. The points indicate mean unsmoothed high-temperature values, as they correspond to successive differences in the column of mean heat contents of table 3 but embodying the small curvature corrections indicated by eq (6).

## 5. Discussion of Experimental Results

Evidence as to the probable accuracy of the heat-content values given by eq (1) and of heat capacity given by the derivative of this equation can be obtained from three sources: (1) The reproducibility or precision of the measurements, (2) an examination of the likely systematic errors, and (3) the agreement among different observers.

Taking into account only the effect of the precision at a given temperature in the individual runs on the empty container and also those on the container with sample, the probable error of the mean unsmoothed net heat content of thorium dioxide sample 1 at a given temperature, relative to that at 0 °C, can be shown from table 3 to average ±0.004 cal g^−1^. This corresponds to a variation from ±0.10 percent or less between 50 and 300 °C to ±0.01 percent or less from 400 to 900 °C. The average deviation of the mean experimental heat-content values from the values given by eq (1) is ±0.014 cal g^−1^. This corresponds to a variation from ±0.21 percent up to and including 300 °C to ±0.04 percent between 400 and 900 °C. The reproducibility of the ice calorimeter was as good during the thorium-dioxide measurements as with the most precise measurements with the same apparatus in the past. A careful analysis of all possible sources of error, systematic as well as accidental, led to the conclusion that the largest uncertainty is that due to the amount of sodium present in the thorium-dioxide sample. The qualitative spectrochemical analysis reported in section 2 indicated less than 0.05 percent sodium, but even this upper limit contributes an uncertainty of ±0.2 percent in both heat-content and heat-capacity values. All other possible sources of error were estimated to contribute ±0.05 percent at 400 °C and ±0.1 percent at 900 °C. Therefore the total probable error in heat-content and heat-capacity values reported here is estimated to be approximately ±0.2 percent between 400 and 900 °C. Below 400 °C the uncertainties in heat content are probably closer to ±0.3 or ±0.4 percent, owing in large part to the decreased percentage reproducibility of the calorimeter because of low heat contents. Measurements of the heat-content of standard-sample *α*-aluminum oxide, made immediately after the present thorium dioxide experiments, indicate agreement at 400 and 700 °C with smoothed reported values [5] within the estimated accuracy of the latter.

The heat-content values obtained for low-density thorium dioxide sample 2 have not been included in this discussion. Because sample 1 was much closer to the X-ray density, had more reproducible heat-content values, and did not show as good agreement with sample 2 as had been expected, the heat-content values of sample 2 were neglected. A complete series of heat-content measurements, not recorded in this paper, was made on thorium dioxide samples 1 and 2 before the reported results were obtained. The subsequent discovery of a leak in the mercury line of the ice calorimeter indicated the advisability of repeating the measurements after the leak was repaired. The results reported here for sample 1 agree well with those obtained earlier, with the exception of those at 200 and 900 °C, which were both higher in the earlier than in the later series of measurements. The measurements on sample 2 made before the leak was repaired indicated that this sample, in general, showed a higher heat content than sample 1 at all temperatures. This trend is indicated by a comparison of the heat-content values in the second and third columns of table 3 although the values agree within the poorer precision on sample 2. This lower precision seems to be due to the sample itself and not to the experimental proceedure.

**Figure 2:**
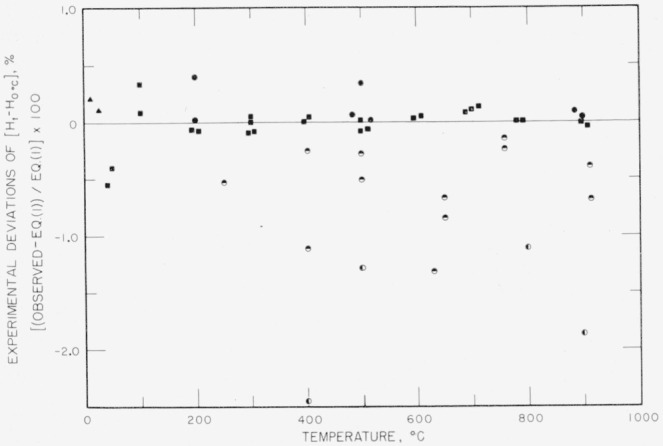
Comparison of the heat content, relative to 0 °C, of thorium dioxide obtained from equation 1 with the values obtained in other investigations. (Some of the observed points have been displaced horizontally by small amounts in order to avoid the confusion of overlapping. For each of the two sets of NBS data, all observed points for a given temperature are based on the *mean* empty-container value observed for that temperature.) -------------, NBS equation 1; ■, NBS sample 1; ●, NBS sample 2; ▲, Osborne and Westrum; ◓, Southard; ◐, Jaeger and Veenstra.

Figure 2 affords a comparison of the present measurements with previously reported values, in the form of percentage deviation of individual unsmoothed measurements of $\left(H_{t}^{o}-H_{0\;{}^{\circ}C}^{o}\right)$ from eq (1). The present measurements fill a previous gap in ThO_2_ heat-content data from 25 to 250 °C. The agreement with Osborne and Westrum is outstanding considering the very small values of heat content involved. Southard [7] used the same form of equation as eq (1) to represent his results, but some of his individual heat-content values deviate from his equation by comparatively large amounts. Southard’s smoothed results above 500 °C are nearly parallel to eq (1), indicating nearly the same heat-capacity values from 500 to 900 °C as obtained in the present work. Because figure 2 represents percentage deviations, this fact is not immediately apparent. Jaeger and Veenstra [8] obtained heat-content values which vary from eq (1) by comparatively large, irregular amounts. Because of the superior precision of the present measurements and previous experience with this calorimeter, the authors believe that the smoothed values in this paper are the most accurate yet reported for ThO_2_ in the high-temperature region.

## 6. Summary

This paper has presented precise measurements of the heat content of high-density sintered thorium dioxide. As anticipated, no transitions which might mar the suitability of this material as a heat-capacity standard were found to occur below 900 °C. Measurements on a lower-density sample showed poorer precision than those on the high-density sample. The two sets of measurements agree within the precision of the former.

Further precise studies should be made to determine the thermal behavior of high-purity ThO_2_ powder and of high-purity samples obtained independently of the batch described in this paper. The authors believe that further heat measurements on pure thorium dioxide should be made to the highest possible temperatures to determine its suitability as a heat-capacity standard for temperatures which exceed the useful range of *α*-Al_2_O_3_. If thorium dioxide is to be used as a standard it should be useful as such from room temperature to temperatures in excess of 2,000 °K.

An important advantage of *α*-Al_2_O_3_ as a standard is the uniformity of the macroscopic crystals used. Macrocrystalline ThO_2_ was not available for the measurements described in this paper. If high-density pressed thorium dioxide is to be a suitable standard it must be shown that different specimens of the same purity and of approximately the same density are close enough in thermal behavior that differences are undetectable within the precision of the best high-temperature calorimetric apparatus.

One important condition which *α*-Al_2_O_3_ is assumed to meet as a heat-capacity standard is that different specimens from the same large batch have nearly identical thermal properties. This assumption has generally been accepted, although the National Bureau of Standards has published data on only two specimens [1,9]. Several other specimens have been checked at one or two temperatures only without observing any significant differences from published values. Such a test is frequently used to monitor the performance of the apparatus used in the present work (see sec 5 of this paper).

Measurements at 600 and 900 °C were made on a second high-density ThO_2_ specimen from the same batch of powder. At the same temperature, all measurements on both specimens agreed within 0.1 percent of the heat content relative to 0 °C. Although this does not definitely prove the suitability of pressed samples as standards, it does support the authors’ belief that within the precision of present-day high-temperature calorimetric measurements, individually pressed, fired, and sintered specimens from the same homogeneous batch of powder should have the same reliability as heat-capacity standards as the macrocrystalline *α*-Al_2_O_3_ now used.

## Figures and Tables

**Table 1:** Impurities in the samples of thorium dioxide^a^

Element	Sample 1	Sample 2	Lower limit of detection
			
	*Weight* %	*Weight* %	*Weight* %
Ag	………(?)	………(?)	……………
Al	0.01	0.008	……………
As	……………	……………	0.01
Au	……………	……………	.0001
B	b<.001	b<.001	.0005
Ba	……………	……………	……………
Be	……………	……………	.0002
Bi	……………	……………	.001
Ca	.005	.005	……………
Cd	……………	……………	……………
Ce	……………	……………	……………
Co	……………	……………	.001
Cr	<.0005	<.0005	.0002
Cu	.005	.003	……………
Fe	.004	.001_5_	……………
Ga	……………	……………	……………
Ge	……………	……………	.0001
Hf	……………	……………	……………
Hg	……………	……………	.001
In	……………	……………	.0001
Ir	……………	……………	……………
La	……………	……………	……………
Mg	<0.0002	<0.0002	0.00005
Mn	<.0005	<.0005	.0001
Mo	……………	……………	.02
Nb	……………	……………	……………
Ni	.009	.007	……………
P	……………	……………	.01
Pb	.0001	.0002_5_	……………
Pt	……………	……………	……………
Sb	……………	……………	.0002
Si	.003	.003_5_	……………
Sn	……(?)	………(?)	.001
Sr	……………	……………	.005
Ta	……………	……………	……………
Ti	……………	……………	.01
Tl	……………	……………	.0001
U	……………	……………	.1
V	……………	……………	.01
W	……………	……………	……………
Y	……………	……………	……………
Yb	……………	……………	……………
Zn	……………	……………	.002
Zr	……………	……………	……………

a A dash indicates that the element was not detected.

b The other elements were determined from specimens ground in a boron carbide mortar. The estimates of boron were made from separate specimens not so treated.

**Table 2:** Individual heat measurements on an empty container

Furnace temperature, *t*	Measured heat	Mean heat
		
*°C*	*cal*	*cal*
50.00	$\{\;\begin{array}{c} 36.61\\ 36.61 \end{array}$	} 36.61
100.00	$\{\;\begin{array}{c} 73.62\\ 73.63 \end{array}$	} 73.62
200.00	$\{\;\begin{array}{c} 149.35\\ 149.79 \end{array}$	} 149.57
300.00	$\{\;\begin{array}{c} 227.87\\ 228.10 \end{array}$	} 227.98
400.00	$\{\;\begin{array}{c} 306.99\\ 306.86 \end{array}$	} 306.92
500.00	$\{\;\begin{array}{c} 387.56\\ 387.71 \end{array}$	} 387.64
600.00	$\{\;\begin{array}{c} 469.79\\ 469.60 \end{array}$	} 469.70
700.0	$\{\;\begin{array}{c} 554.20\\ 554.60 \end{array}$	} 554.40
800.0	$\{\;\begin{array}{c} 641.94\\ 642.21 \end{array}$	} 642.08
900.0	$\{\;\begin{array}{c} 731.04\\ 730.99 \end{array}$	} 731.02

**Table 3:** Relative heat content of thorium dioxide, (*H_t_–H_0_ °c*)

Furnace temperature, *t*	Individual heat-content measurements		Mean sample 1	Calculated eq. (1)	Mean sample 1 minus calculated
	Sample 1	Sample 2			
					
*° C*	*cal g*^−1^	*cal g*^−1^	*cal g*^−1^	*cal g*^−1^	*cal g*^−1^
50.00	$\{\;\begin{array}{c} 2.780\\ 2.776 \end{array}$		} 2.778	2.792	−0.014
100.00	$\{\;\begin{array}{c} 5.763\\ 5.749 \end{array}$		} 5.756	5.745	+.011
200.00	$\{\;\begin{array}{c} 11.946\\ 11.944 \end{array}$	11.999 11.953	} 11.945	11.954	−.009
300.00	$\{\;\begin{array}{c} 18.428\\ 18.399\\ 18.401 \end{array}$		} 18.409	18.420	−.011
400.00	$\{\;\begin{array}{c} 25.056\\ 25.066 \end{array}$		} 25.061	25.058	+.003
500.00	$\{\;\begin{array}{c} 31.810\\ 31.813\\ 31.827 \end{array}$	31.928 31.841 31.826	} 31.817	31.829	−.012
600.00	$\{\;\begin{array}{c} 38.718\\ 38.722 \end{array}$		} 38.720	38.709	+.011
700.0	$\{\;\begin{array}{c} 45.727\\ 45.737\\ 45.716 \end{array}$		} 45.727	45.687	+.040
800.0	$\{\;\begin{array}{c} 52.754\\ 52.748 \end{array}$		} 52.751	52.755	−.004
900.0	$\{\;\begin{array}{c} 59.888\\ 59.876 \end{array}$	59.914 59.940	} 59.882	59.906	−.024

**Table 4:** Thermodynamic properties of thorium dioxide

*T*	$C_{p}^{o}$	$H_{T}^{o}-H_{0{\;}^{{}^{\circ}}K}^{o}$	$S_{T}^{o}-S_{0{\;}^{{}^{\circ}}K}^{o}$	$-\frac{F_{T}^{o}-H_{0{\;}^{{}^{\circ}}K}^{o}}{T}$
				
*°K*	*cal/mole-deg*	*cal/mole*	*cal/mole-deg*	*cal/mole-deg*
298.15	14.76	2524.4	15.593	7.126
300	14.81	2551.6	15.683	7.178
320	15.18	2851.6	16.651	7.740
340	15.50	3158.4	17.581	8.292
360	15.77	3471.1	18.474	8.834
380	16.00	3788.9	19.334	9.363
400	16.21	4111.1	20.160	9.882
420	16.39	4437.1	20.955	10.390
440	16.55	4766.6	21.721	10.888
460	16.70	5099.2	22.461	11.376
480	16.84	5434.6	23.174	11.852
500	16.96	5772.5	23.864	12.319
550	17.22	6627.2	25.493	13.444
600	17.44	7494.0	27.001	14.510
650	17.64	8371.1	28.405	15.526
700	17.81	9257.4	29.719	16.494
750	17.97	10151.9	30.953	17.417
800	18.11	11053.9	32.117	18.299
850	18.25	11962.9	33.219	19.145
900	18.37	12878.4	34.266	19.957
950	18.50	13800.2	35.263	20.736
1000	18.61	14728.0	36.214	21.486
1050	18.73	15661.6	37.125	22.209
1100	18.84	16600.7	37.999	22.907
1150	18.95	17545.4	38.838	23.582
1200	19.05	18495.3	39.648	24.235

## Footnotes

2 Figures in brackets indicate literature references at the end of this paper.

3 To grind the thorium dioxide specimens in preparation for their analysis, mills of different composition (boron carbide, alundum, and plastic) were used to check the possibility of appreciable contamination. Of the nine specimens analyzed, the two which had been ground in alundum showed fifteen times as much silicon (0.05 percent) as the others, and were discounted. This difference in silicon would correspond to two specimens of ThO_2_ whose relative heat contents at the same temperature should differ by approximately 0.5 percent; however, several thermal measurements made on both types of specimen indicated an actual difference of 0.05±0.05 percent. There was no discrepancy with respect to the percentage of any other chemical element.

4 Temperatures are on the International Temperature Scale of 1948 as modified in 1954. This assigns the temperature 273.16 °K to the triple point of water and defines zero on the Celsius scale as 0.0100 degree below this [3].
